# Dualistic Dynamics in Neuropsychiatry: From Monoaminergic Modulators to Multiscale Biomarker Maps

**DOI:** 10.3390/biomedicines13061456

**Published:** 2025-06-13

**Authors:** Masaru Tanaka, Simone Battaglia

**Affiliations:** 1 Danube Neuroscience Research Laboratory, HUN-REN-SZTE Neuroscience Research Group, Hungarian Research Network, University of Szeged (HUN-REN-SZTE), Tisza Lajos krt. 113, H-6725 Szeged, Hungary; 2 Center for Studies and Research in Cognitive Neuroscience, Department of Psychology “Renzo Canestrari”, Cesena Campus, Alma Mater Studiorum, University of Bologna, 47521 Bologna, Italy; simone.battaglia@unibo.it; 3 Department of Psychology, University of Turin, 10124 Turin, Italy

## 1. Introduction: The Dualistic Lens

Neuropsychiatry lives at the crossroads of chemistry and cognition, where millisecond synaptic sparks sculpt decades-long stories of mood, memory, and identity [1,2,3]. The same organ then turns inward, making brain–body self-inquiry a central paradox now probed by advanced imaging, stimulation, and physiological modeling [4,5,6]. Modern data depict neurotransmission as a yin–yang choreography: glutamatergic bursts checked by gamma-aminobutyric acid (GABA) brakes and cortical volleys echoed by visceral afferents carrying gut, liver, and immune cues, while nanoscopic receptor tweaks reverberate through whole networks [7,8,9]. Mapping the harmonics of this loop may decode consciousness and its breakdown across neuropsychiatric and neurodegenerative disease, since equilibrium failure begets illness [10,11,12].

Within this framework, psychiatric and neurodegenerative disorders appear less like single-node breakdowns and more like systemic disequilibria [13,14,15]. When the delicate balance collapses, as in post-traumatic stress disorder (PTSD), dysregulated fear circuitry and hypothalamic–pituitary–adrenal (HPA) axis perturbations create neuroendocrine noise—cortisol volatility, monoaminergic surges, failed fear extinction, and intrusive memory loops [16,17,18]. Similarly, in Alzheimer’s disease (AD) or Parkinson’s disease, oxidative stress derails networks, yet antioxidant-rich diets and compounds modestly stabilize memory and action control in longitudinal human cohorts [17,19,20]. Complementing those longitudinal data, reviews show that phytocompounds—including polyphenols, alkaloids, and terpenoids—improve cognition and neuropsychiatric symptoms across AD and related disorders [21,22,23]. An expanding Topical Collection, “Neurodegeneration No More,” now assembles cutting-edge diagnostics, therapeutic targets, and integrative care models aimed at reversing disease trajectories, underscoring the urgency of interdisciplinary collaboration for neurodegenerative disease management [24]. Reinforcing this perspective, an increasing amount of evidence maps how medicinal plants inhibit nuclear factor kappa-light-chain-enhancer of activated B cells (NF-κB), cyclooxygenase-2 (COX-2), and inducible nitric oxide synthase (iNOS) while activating nuclear factor erythroid 2-related factor 2 (Nrf2) signaling, detailing dose windows and translational gaps across progressive neurodegenerative disease [25,26,27]. Nonetheless, three enduring translational gaps impede the conversion of conceptual advances into clinical benefit. The first is the bench-to-bedside divide: antioxidant interventions that show efficacy in rodent models or in silico screens rarely advance to well-powered clinical trials that rigorously account for sex, age, lifestyle factors, and comorbidities [27,28,29]. Second, a network-integration blind spot: investigations isolate single receptors, overlooking the multiplex cortisol–monoamine–immune crosstalk that governs resilience across organs and lifespan [30,31,32]. Third, the composite-biomarker vacuum: p-tau isoforms, circulating microRNAs, cortisol rhythms, or receptor-binding positron emission tomography (PET) signals remain unharmonized, limiting our ability to stratify patients, forecast trajectories, and personalize interventions [33,34,35,36,37].

This Special Issue, “Dualistic Equilibrium in Neurotransmission and Beyond,” is curated to illuminate that landscape, bridge its chasms, and chart future routes “https://www.mdpi.com/journal/biomedicines/special_issues/Neurotransmission (accessed on 12 June 2025)”. By uniting eight studies that journey from resveratrol-induced monoamine oxidase A (MAO-A) allostery and trace amine receptor agonism to orexin-orchestrated stress adaptation and isoform-specific tau diagnostics, the collection strives to translate basic insights, weave an empirically grounded system map, and lay scaffolds for next-generation composite biomarker panels. Together, these contributions aim to ignite interdisciplinary alliances spanning medicinal chemistry, network neuroscience, computational modeling, clinical psychiatry, policy makers, caregivers, and public health leaders worldwide, driving progress in basic science, clinical translation, and population health and steering lasting therapeutic innovation for patients across the globe.

## 2. Eight Windows on Equilibrium

Molecular docking, dynamics, and predator-stressed rats reveal resveratrol and its glucuronide occupy an allosteric pocket on MAO-A, dampening brain and liver enzyme activity and pointing dietary polyphenols toward anxiolytic monoaminergic therapy [38]. Shemiakova et al. synthesize evidence positioning trace amine-associated receptors (TAARs) as novel antidepressant targets [39] (Table 1). TAAR1–TAAR9 extend beyond smell, populating limbic circuits. The TAAR1 agonist ulotaront succeeds in phase 2/3 depression trials. Preclinical data show that TAAR2/TAAR5 shape emotion, monoamine signaling, and hippocampal neurogenesis, suggesting TAAR-targeted drugs could outpace monoamine therapies and cut side-effects in hard-to-treat depression populations.

Perinatal exposure of rats to 5-hydroxytryptophan (5-HTP) or tranylcypromine induces adult-onset histomorphometric and metabolic alterations in the jejunum and liver, spotlighting how early hyperserotonemia imprints a persistent gut–liver serotonergic loop beyond the brain [40]. Pharmacological pairing of CB1 and 5-HT1A agonists or antagonists before versus after cold stress in rats showed serotonergic signaling maintains stress-induced analgesia pre-stress while cannabinoid modulation predominates post-stress, exposing time-specific crosstalk vital for designing anti-stress pain interventions [41]. A narrative review synthesizes evidence that orexin/hypocretin neurons act as master neuromodulators coupling vigilance with autonomic, endocrine, and behavioral stress responses; highlights their roles in fear, anxiety, and learning; and surveys emerging orexin-based pharmacotherapies for sleep and stress disorders [42].

In 111 outpatients with schizophrenia, higher disorganized and obsessive–compulsive symptom scores independently predicted greater depressive severity, whereas a longer duration of untreated psychosis paradoxically correlated with milder depression, indicating that early circuit disorganization, rather than demographic factors, is the primary driver of comorbid depressive burden [43]. Striatal injection of a miR-200b-3p antagomir in spontaneously hypertensive rats alleviated inattention, reduced pro-inflammatory cytokines, and elevated antioxidant enzymes, spotlighting miR-200b-3p as a promising therapeutic microRNA target for attention-deficit/hyperactivity disorder (ADHD) [44].

A narrative review of 85 original studies concludes that plasma and cerebrospinal fluid (CSF) p-tau217 and p-tau231 outperform p-tau181 in detecting preclinical Aβ pathology, track tau-tangle progression, and differentiate AD from other dementias, positioning these isoform-specific assays as front-line, minimally invasive biomarkers for early diagnosis and staging [45].

## 3. Bridging the Gaps: How These Papers Move the Needle

Bridging conceptual insights to clinical utility requires proof that discoveries travel the full distance from molecules to networks to patients. The following three subsections illustrate this trajectory, showing how preclinical candidates acquire translational momentum, how receptor-centric views expand into circuit logic, and how layered biomarkers converge into actionable precision panels.

### 3.1. Translational Momentum of Rodent Leads

Three rodent-based investigations within the Special Issue push molecular leads toward the clinic. In chronically predator-stressed rats, resveratrol and its glucuronide occupied a newly charted monoamine-oxidase-A allosteric pocket, halved cortical and hepatic enzyme activity, and attenuated anxiety-like behavior, suggesting that a safe nutritional polyphenol could be repurposed for affective disorders [38]. Complementing this single-compound result, a recent narrative review shows that phytochemicals—from curcumin to flavonoids and alkaloids—concurrently quell neuroinflammation, oxidative stress, and mitochondrial dysfunction, easing major-depression phenotypes in preclinical and clinical studies [46]. Systemic delivery of the selective trace amine receptor-1 agonist RO5263397 reversed immobility in forced-swim and tail-suspension tests, normalized hippocampal neurogenesis, and reduced peripheral corticosterone, mechanistic outcomes echoed by the clinical-stage compound ulotaront now in phase 2/3 trials for depression and anxiety [39]. Meanwhile, CRISPR/Cas9 knockout of kynurenine-aminotransferase genes in mice suppressed cerebellar and hippocampal oxidative phosphorylation, highlighting kynurenine aminotransferase-dependent kynurenic control of mitochondrial respiration and energy metabolism in vivo [47,48]. Extending that axis, a quinoline-focused narrative review shows how halogenation, esterification, and in silico screening refine tryptophan-derived metabolites into multi-target ligands that cross the blood–brain barrier and quell excitotoxic-immune cascades, steering rational design for next-generation quinoline-based mitochondrial therapeutics [49]. Finally, stereotaxic inhibition of striatal miR-200b-3p in spontaneously hypertensive rats restored attentional performance, suppressed interleukin (IL)-6 and tumor necrosis factor (TNF)-alpha (α), and boosted superoxide-dismutase activity, delineating a microRNA–inflammation axis ripe for biomarker-guided antisense therapies that are advancing into first-in-human safety assessments [44]. Collectively, these convergent results validate target engagement, demonstrate behavioral rescue across independent models, and furnish biochemical read-outs that align seamlessly with ongoing or planned human studies, thereby shortening the bench-to-bedside journey.

### 3.2. From Receptor Islands to Circuit Continents—System-Level Integration

Perinatal elevation of systemic serotonin through 5-HTP or tranylcypromine re-sculpted adult gut–liver physiology: villus shortening, crypt hyperplasia, suppressed enterochromaffin 5-HT staining, and altered hepatic 5-HT-metabolizing enzymes, establishing a persistent peripheral serotonin circuit poised to influence brain mood circuits via the portal vein and vagus [40]. In an acute cold-stress model, bidirectional pharmacology revealed time-stamped reciprocity between CB1 and 5-HT1A signaling: co-agonism before stress magnified analgesia, whereas the identical cocktail after stress dampened it; selective antagonist pairings confirmed serotonergic dominance in stress initiation and cannabinoid control during recovery, underscoring a dynamic receptor handshake across limbic, periaqueductal-gray, and HPA nodes [41]. Complementing these rodent data, a comprehensive review positions orexin neurons as master switches that broadcast stress salience through widespread projections, coordinating vigilance, autonomic, and endocrine outputs while modulating monoamines, endocannabinoids, and neuropeptides, thereby offering a top-down scaffold for multi-target drug design [42]. Collectively, these findings shift the narrative from single-receptor pharmacology to network neuroscience, illuminating organ-to-brain loops and temporal hierarchies that future therapeutics must respect. A recent synthesis highlights that age-related vascular dysfunction, sarcopenic muscle loss, and neurodegenerative cognitive decline constitute a pathophysiological triad unified by oxidative stress and chronic inflammation [50].

### 3.3. Toward Composite Precision Panels

The Special Issue showcases how disparate biomarker layers can converge into patient-stratifying toolkits. A narrative synthesis of 85 studies reports that plasma and CSF p-tau217 and p-tau231 consistently outclass p-tau181, flagging pre-amyloid pathology with >90% accuracy, tracking Braak staging, and cleanly separating AD from frontotemporal and vascular dementias [45]. In a rodent model of ADHD, stereotaxic silencing of striatal miR-200b-3p rescued working-memory deficits, lowered IL-6 and TNF-α, and restored superoxide-dismutase activity, positioning this microRNA as both a mechanistic node and a peripheral read-out for neuro-immune dysregulation in ADHD [44]. Complementing these fluid signatures, a clinical cohort of 111 individuals with schizophrenia showed that disorganized and obsessive–compulsive symptom clusters, rather than demographic variables, predicted depressive load, while a longer untreated psychosis interval paradoxically attenuated it, nominating symptom network topology as a low-cost behavioral biomarker [43]. Together, isoform-specific tau assays, actionable microRNAs, and data-driven symptom lattices foreshadow multiplex panels capable of early detection, mechanistic staging, and personalized therapeutic steering across neuropsychiatric spectra.

## 4. Future Frontiers

Translating the mechanistic advances documented in this Special Issue into clinically transformative applications demands an integrated research agenda. We therefore outline five strategic imperatives—ranging from multi-omics imaging convergence to ethical governance—that together promise to consolidate molecular discoveries, refine patient stratification across the lifespan, and guide the deployment of network-targeted interventions.

### 4.1. Multi-Omics Coupling with In Vivo Imaging

Next-generation discovery hinges on stitching lipidomic, metabolomic and receptor-specific PET readouts within individuals [51,52,53]. Chemo-connectome scans link arachidonic acid flux or phospholipid turnover to network fragility [54,55,56,57]. To deepen interpretability, pipelines should also track brain-autonomic synchrony, capturing covariation between cortical activity and cardiac deceleration, charting loops that tie emotions to autonomic outflow [4,52,55]. Delivering this vision will require hybrid scanners, biofluid taps, and cloud analytics that fuse terabytes of omics spectra with voxel-wise binding maps in time [58,59,60].

### 4.2. Longitudinal, Sex-Balanced Cohorts

Most existing datasets are cross-sectional snapshots or male-skewed rodent lines [61,62,63]. We need cradle-to-senescence cohorts, stratified by chromosomal and hormonal sex, that collect neuroimaging, multi-omics, immune read-outs, and detailed life-event chronologies at repeated milestones [64,65,66]. Tracking the same individuals from perinatal stages through puberty, reproductive transitions, and neurodegenerative risk windows will reveal when gut–liver–brain loops or monoaminergic balances hit irreversible tipping points—and whether those inflections differ by sex, ancestry, or socio-environmental load [67,68,69,70,71].

### 4.3. Digital Phenotyping and Artificial Intelligence (AI) Biomarker Fusion

Wearables, smartphones, and ambient sensors convert gait micro-variability, sleep architecture, voice prosody, and social-touch signatures into continuous phenomic streams, illustrating the field’s pivot from serendipitous drug discovery toward precision mental health research [72,73,74,75,76]. Parallel conceptual work urges replacing categorical Diagnostic and Statistical Manual of Mental Disorders (DSM)/International Classification of Diseases (ICD) diagnoses with dimensional Hierarchical Taxonomy of Psychopathology (HiTOP) and Research Domain Criteria (RDoC) taxonomies, integrating these phenomic streams into empirically anchored, biologically informed nosology [77,78,79,80,81]. Coupled with federated artificial intelligence (AI) that simultaneously ingests PET-omics, microRNA panels, and symptom lattices, these data lakes could generate personalized risk curves updated hourly [82,83,84,85,86]. Key priorities include open ontologies for feature harmonization, self-supervised algorithms that learn across modalities with minimal labels, and privacy-preserving architectures that keep raw data on-device while sharing encrypted embeddings for population-level modeling [87,88].

### 4.4. Network-Level Interventions

Insights from circuit-centric papers invite therapies that modulate entire networks rather than single receptors [89,90,91]. Closed-loop deep-brain or vagus nerve stimulators, guided by real-time neurochemical sensors, could nudge maladaptive oscillations back into equilibrium [89,90,91]. On the peripheral front, liver-targeted MAO-A inhibitors or gut-restricted 5-HT modulators may recalibrate central affect without crossing the blood–brain barrier, reducing systemic side-effects [68,92,93]. Combinatorial designs—such as pairing orexin antagonists with anti-inflammatory microRNA mimics—should be tested in adaptive platform trials that can rapidly prune ineffective arms [94,95,96,97].

### 4.5. Ethical and Regulatory Horizon-Scanning

As datasets sprawl and interventions become closed-loop, guardrails must keep pace [98,99,100]. Regulators will need new guidelines for multi-omics companion diagnostics, AI-driven adaptive trials, and implantable devices that learn on the fly [101,102,103]. Equitable access requires subsidized genomic and imaging pipelines in low-resource settings and bias audits for machine learning models [100,101,102]. Finally, consent frameworks should allow participants granular control over which data layers—lipids, speech, location—enter shared repositories, ensuring progress does not outstrip public trust [98,99,103].

## 5. Conclusions: Toward a Convergent Neuropsychiatry

The studies collected in this Special Issue validate “dualistic equilibrium” as a unifying framework for neuropsychiatric science, showing that health depends on balanced interactions between excitation and inhibition, central and peripheral signaling, and molecular events and network dynamics. Each contribution shifts the discourse from isolated observations to a layered map that connects monoaminergic allostery, trace amine receptor pharmacology, orexin-regulated stress circuitry, microRNA-driven inflammation, and isoform-specific tau pathology. The resulting atlas is mechanistically precise, since it identifies actionable binding pockets, temporal receptor handshakes, and fluid biomarkers; translationally actionable, because several rodent leads already align with phase 2 or phase 3 trials; and ethically responsible, thanks to an explicit focus on sex-balanced cohorts, privacy-aware digital phenotyping, and equitable access to advanced diagnostics.

The next milestone is collective execution. Specialist silos must give way to consortia that integrate medicinal chemistry, imaging physics, multi-omics analytics, computational psychiatry, regulatory science, and patient advocacy. Shared protocols for hybrid PET–omics pipelines, harmonized outcome measures for adaptive trials, and interoperable data standards for wearable-derived phenomics will accelerate discovery while preventing duplication. Funding bodies should prioritize platforms that enroll cradle-to-senescence participants in diverse settings, ensuring that findings generalize across sex, ancestry, and socio-economic strata. Regulatory agencies can support this trajectory by creating pathways for composite biomarker approval and real-time neuromodulation devices.

Anchored in dualistic equilibrium, guided by a systems lens, and propelled by an ethic of inclusivity, the field is now positioned to transform fragmented mechanistic snapshots into integrated, patient-centered care paradigms. Progress will hinge not on isolated breakthroughs but on coordinated, transparent collaboration that keeps scientific ambition and societal responsibility in steady alignment.

## Figures and Tables

**Table 1:** Thematic clustering of the eight contributions in the Special Issue “Dualistic Equilibrium in Neurotransmission and Beyond.” The table groups each paper under a shared mechanistic theme, lists its concise title, and links to the corresponding reference segment. This layout spotlights how the collection spans monoaminergic modulation, serotonergic/peripheral stress circuits, symptom-level and molecular modulators in psychiatric disorders, and biomarker discovery for neurodegeneration.

Group/Topic	Shared Idea	Paper	Ref.
Monoaminergic Targets for Mood Regulation	MAO-A or TAARs rebalance monoamines	Resveratrol as MAO-A allosteric modulator	[38]
		TAARs as novel antidepressant targets	[39]
Serotonergic and Peripheral Stress Systems	5-HT, CB1, orexin in stress and organs	Perinatal 5-HT enhancers alter gut–liver axis	[40]
		CB1–5-HT1A in stress-induced analgesia	[41]
		Orexin system in stress vigilance	[42]
Symptom Dynamics in Brain Disorders	Clinical/microRNA modulators of symptoms	OCD symptoms worsen depression in schizophrenia	[43]
		miR-200b-3p antagonism reduces ADHD traits	[44]
Molecular Biomarkers for Neurodegeneration	Next-gen fluid markers for early AD diagnosis	Plasma p-tau217/231 differentiation of AD	[45]

AD, Alzheimer’s disease; ADHD, attention-deficit/hyperactivity disorder; CB1, cannabinoid receptor type 1; 5-HT, 5-hydroxytryptamine; 5-HT1A, 5-hydroxytryptamine receptor type 1A; miR-200b-3p, microRNA 200b family subtype the 3′ strand; MAO-A, monoamine oxidase A; OCD, obsessive–compulsive disorder; p-tau217/231, phosphorylated tau protein at threonine 217 and threonine 231; TAARs, trace amine-associated receptors.

## Abbreviations

The following abbreviations are used in this manuscript:

AD	Alzheimer’s disease
ADHD	attention-deficit/hyperactivity disorder
AI	artificial intelligence
CB1	cannabinoid receptor type 1
CSF	cerebrospinal fluid
HPA	hypothalamic–pituitary–adrenal
5-HT	5-hydroxytryptamine
5-HT1A	5-hydroxytryptamine receptor type 1A
5-HTP	5-hydroxytryptophan
IL	interleukin
MAO-A	monoamine oxidase A
miR-200b-3p	microRNA 200b family subtype the 3′ strand
OCD	obsessive–compulsive disorder
PET	positron emission tomography
p-tau217/231	phosphorylated tau protein at threonine 217 and threonine 231
TAARs	trace amine-associated receptors
TNF	tumor necrosis factor

## References

[1] Rabl M., Clark C., Dayon L., Popp J. (2025). Neuropsychiatric symptoms in cognitive decline and Alzheimer’s disease: Biomarker discovery using plasma proteomics. J. Neurol. Neurosurg. Psychiatry.

[2] Pontone G.M., Mills K.A., Smith G.S. (2017). Molecular imaging in neuropsychiatry. Int. Rev. Psychiatry.

[3] Milham M.P., Craddock R.C., Klein A. (2017). Clinically useful brain imaging for neuropsychiatry: How can we get there?. Depress. Anxiety.

[4] Battaglia S., Servajean P., Friston K.J. (2025). The paradox of the self-studying brain. Phys. Life Rev..

[5] Dary Z., Lenggenhager B., Lagarde S., Medina Villalon S., Bartolomei F., Lopez C. (2023). Neural bases of the bodily self as revealed by electrical brain stimulation: A systematic review. Hum. Brain Mapp..

[6] Bradley C., Nydam A.S., Dux P.E., Mattingley J.B. (2022). State-dependent effects of neural stimulation on brain function and cognition. Nat. Rev. Neurosci..

[7] Zuurbier K.R., Fonseca R.S., Arneaud S.L., Wall J.M., Kim J., Tatge L., Otuzoglu G., Bali S., Metang P., Douglas P.M. (2024). Yin Yang 1 and guanine quadruplexes protect dopaminergic neurons from cellular stress via transmissive dormancy. Nat. Commun..

[8] Gupta R., Advani D., Yadav D., Ambasta R.K., Kumar P. (2023). Dissecting the Relationship Between Neuropsychiatric and Neurodegenerative Disorders. Mol. Neurobiol..

[9] Novellino F., Saccà V., Donato A., Zaffino P., Spadea M.F., Vismara M., Arcidiacono B., Malara N., Presta I., Donato G. (2020). Innate Immunity: A Common Denominator between Neurodegenerative and Neuropsychiatric Diseases. Int. J. Mol. Sci..

[10] Luppi A.I., Vohryzek J., Kringelbach M.L., Mediano P.A., Craig M.M., Adapa R., Carhart-Harris R.L., Roseman L., Pappas I., Peattie A.R. (2023). Distributed harmonic patterns of structure-function dependence orchestrate human consciousness. Commun. Biol..

[11] Wamsley B., Geschwind D.H. (2020). Functional genomics links genetic origins to pathophysiology in neurodegenerative and neuropsychiatric disease. Curr. Opin. Genet. Dev..

[12] Cummings J. (2021). The Role of Neuropsychiatric Symptoms in Research Diagnostic Criteria for Neurodegenerative Diseases. Am. J. Geriatr. Psychiatry.

[13] Finlay S., Rudd D., McDermott B., Sarnyai Z. (2022). Allostatic load and systemic comorbidities in psychiatric disorders. Psychoneuroendocrinology.

[14] Du X., Pang T.Y. (2015). Is Dysregulation of the HPA-Axis a Core Pathophysiology Mediating Co-Morbid Depression in Neurodegenerative Diseases?. Front. Psychiatry.

[15] Michopoulos V., Vester A., Neigh G. (2016). Posttraumatic stress disorder: A metabolic disorder in disguise?. Exp. Neurol..

[16] Battaglia S., Di Fazio C., Borgomaneri S., Avenanti A. (2025). Cortisol imbalance and fear learning in PTSD: Therapeutic approaches to control abnormal fear responses. Curr. Neuropharmacol..

[17] Battaglia S., Nazzi C., Thayer J.F. (2024). Genetic differences associated with dopamine and serotonin release mediate fear-induced bradycardia in the human brain. Transl. Psychiatry.

[18] Algamal M., Pearson A.J., Hahn-Townsend C., Burca I., Mullan M., Crawford F., Ojo J.O. (2021). Repeated unpredictable stress and social isolation induce chronic HPA axis dysfunction and persistent abnormal fear memory. Prog. Neuropsychopharmacol. Biol. Psychiatry.

[19] Battaglia S., Nazzi C., Di Fazio C., Borgomaneri S. (2024). The role of pre-supplementary motor cortex in action control with emotional stimuli: A repetitive transcranial magnetic stimulation study. Ann. N. Y. Acad. Sci..

[20] Veurink G., Perry G., Singh S.K. (2020). Role of antioxidants and a nutrient rich diet in Alzheimer’s disease. Open Biol..

[21] de Lima E.P., Laurindo L.F., Catharin V.C.S., Direito R., Tanaka M., Jasmin Santos German I., Lamas C.B., Guiguer E.L., Araújo A.C., Fiorini A.M.R. (2025). Polyphenols, Alkaloids, and Terpenoids Against Neurodegeneration: Evaluating the Neuroprotective Effects of Phytocompounds Through a Comprehensive Review of the Current Evidence. Metabolites.

[22] Can B., Sanlier N. (2025). Alzheimer, Parkinson, dementia, and phytochemicals: Insight review. Crit. Rev. Food Sci. Nutr..

[23] Solfrizzi V., Agosti P., Lozupone M., Custodero C., Schilardi A., Valiani V., Santamato A., Sardone R., Dibello V., Di Lena L. (2018). Nutritional interventions and cognitive-related outcomes in patients with late-life cognitive disorders: A systematic review. Neurosci. Biobehav. Rev..

[24] Tanaka M., Battaglia S., Liloia D. (2025). Navigating Neurodegeneration: Integrating Biomarkers, Neuroinflammation, and Imaging in Parkinson’s, Alzheimer’s, and Motor Neuron Disorders. Biomedicines.

[25] Barbalho S.M., Leme Boaro B., da Silva Camarinha Oliveira J., Patočka J., Barbalho Lamas C., Tanaka M., Laurindo L.F. (2025). Molecular Mechanisms Underlying Neuroinflammation Intervention with Medicinal Plants: A Critical and Narrative Review of the Current Literature. Pharmaceuticals.

[26] Lv H., Ren W., Zheng Y., Wang L., Lu G., Yi P., Ci X. (2016). Tenuigenin exhibits anti-inflammatory activity via inhibiting MAPK and NF-κB and inducing Nrf2/HO-1 signaling in macrophages. Food Funct..

[27] Puppala E.R., Prasad N., Prakash A.N., Abubakar M., Syamprasad N.P., Gangasani J.K., Naidu V.G.M. (2024). Mesua assamica (King & Prain) kosterm. bark ethanolic extract attenuates rheumatoid arthritis via down-regulating TLR4/NF-κB/COX-2/iNOS and activation of Nrf2/HO-1 pathways: A comprehensive study on in-vitro and in-vivo models. J. Ethnopharmacol..

[28] Hanlon P., Butterly E.W., Shah A.S., Hannigan L.J., Lewsey J., Mair F.S., Kent D.M., Guthrie B., Wild S.H., Welton N.J. (2023). Treatment effect modification due to comorbidity: Individual participant data meta-analyses of 120 randomised controlled trials. PLoS Med..

[29] Simu S.Y., Alam M.B., Kim S.Y. (2022). The Activation of Nrf2/HO-1 by 8-Epi-7-deoxyloganic Acid Attenuates Inflammatory Symptoms through the Suppression of the MAPK/NF-κB Signaling Cascade in In Vitro and In Vivo Models. Antioxidants.

[30] Walker F.R., Pfingst K., Carnevali L., Sgoifo A., Nalivaiko E. (2017). In the search for integrative biomarker of resilience to psychological stress. Neurosci. Biobehav. Rev..

[31] de Kloet E.R., Joëls M. (2024). The cortisol switch between vulnerability and resilience. Mol. Psychiatry.

[32] Boahen A., Hu D., Adams M.J., Nicholls P.K., Greene W.K., Ma B. (2023). Bidirectional crosstalk between the peripheral nervous system and lymphoid tissues/organs. Front. Immunol..

[33] Sethi S., Brietzke E. (2016). Omics-based biomarkers: Application of metabolomics in neuropsychiatric disorders. Int. J. Neuropsychopharmacol..

[34] Wesseling H., Mair W., Kumar M., Schlaffner C.N., Tang S., Beerepoot P., Fatou B., Guise A.J., Cheng L., Takeda S. (2020). Tau PTM Profiles Identify Patient Heterogeneity and Stages of Alzheimer’s Disease. Cell.

[35] van den Berg M.M.J., Krauskopf J., Ramaekers J.G., Kleinjans J.C.S., Prickaerts J., Briedé J.J. (2020). Circulating microRNAs as potential biomarkers for psychiatric and neurodegenerative disorders. Prog. Neurobiol..

[36] Ryan R., Booth S., Spathis A., Mollart S., Clow A. (2016). Use of Salivary Diurnal Cortisol as an Outcome Measure in Randomised Controlled Trials: A Systematic Review. Ann. Behav. Med..

[37] Fu H., Rong J., Chen Z., Zhou J., Collier T., Liang S.H. (2022). Positron Emission Tomography (PET) Imaging Tracers for Serotonin Receptors. J. Med. Chem..

[38] Novak J., Tseilikman O.B., Shatilov V.A., Zhukov M.S., Shevyrin V.A., Khismatullina Z.R., Fedorova A.M., Patrikyan G.N., Khaibullin T.L., Tseilikman V.E. (2025). Resveratrol and Its Metabolite as Potential Allosteric Regulators of Monoamine Oxidase A Activity in the Brain and Liver Under Chronic Predator Stress. Biomedicines.

[39] Shemiakova T.S., Efimova E.V., Gainetdinov R.R. (2024). TAARs as Novel Therapeutic Targets for the Treatment of Depression: A Narrative Review of the Interconnection with Monoamines and Adult Neurogenesis. Biomedicines.

[40] Gračan R., Blažević S.A., Brižić M., Hranilovic D. (2024). Beyond the Brain: Perinatal Exposure of Rats to Serotonin Enhancers Induces Long-Term Changes in the Jejunum and Liver. Biomedicines.

[41] Nocheva H., Stoynev N., Vodenicharov V., Krastev D., Krastev N., Mileva M. (2024). Cannabinoid and Serotonergic Systems: Unraveling the Pathogenetic Mechanisms of Stress-Induced Analgesia. Biomedicines.

[42] Jászberényi M., Thurzó B., Bagosi Z., Vécsei L., Tanaka M. (2024). The Orexin/Hypocretin System, the Peptidergic Regulator of Vigilance, Orchestrates Adaptation to Stress. Biomedicines.

[43] Panov G., Dyulgerova S., Panova P., Stefanova S. (2024). Untangling depression in schizophrenia: The role of disorganized and obsessive-compulsive symptoms and the duration of untreated psychosis. Biomedicines.

[44] Chang T.-M., Lin H.-L., Tzang C.-C., Liang J.-A., Hsu T.-C., Tzang B.-S. (2024). Unraveling the Role of miR-200b-3p in Attention-Deficit/Hyperactivity Disorder (ADHD) and Its Therapeutic Potential in Spontaneously Hypertensive Rats (SHR). Biomedicines.

[45] Jarek D.J., Mizerka H., Nuszkiewicz J., Szewczyk-Golec K. (2024). Evaluating p-tau217 and p-tau231 as biomarkers for early diagnosis and differentiation of Alzheimer’s Disease: A narrative review. Biomedicines.

[46] Figueiredo Godoy A.C., Frota F.F., Araújo L.P., Valenti V.E., Pereira E.d.S.B.M., Detregiachi C.R.P., Galhardo C.M., Caracio F.C., Haber R.S., Fornari Laurindo L. (2025). Neuroinflammation and Natural Antidepressants: Balancing Fire with Flora. Biomedicines.

[47] Juhász L., Spisák K., Szolnoki B.Z., Nászai A., Szabó Á., Rutai A., Tallósy S.P., Szabó A., Toldi J., Tanaka M. (2025). The Power Struggle: Kynurenine Pathway Enzyme Knockouts and Brain Mitochondrial Respiration. J. Neurochem..

[48] Tanaka M., Szabó Á., Vécsei L. (2024). Redefining roles: A paradigm shift in tryptophan–kynurenine metabolism for innovative clinical applications. Int. J. Mol. Sci..

[49] Tanaka M., Szatmári I., Vécsei L. (2025). Quinoline Quest: Kynurenic Acid Strategies for Next-Generation Therapeutics via Rational Drug Design. Pharmaceuticals.

[50] de Lima E.P., Tanaka M., Lamas C.B., Quesada K., Detregiachi C.R.P., Araújo A.C., Guiguer E.L., Catharin V.M.C.S., de Castro M.V.M., Junior E.B. (2024). Vascular impairment, muscle atrophy, and cognitive decline: Critical age-related conditions. Biomedicines.

[51] Wang R., Li B., Lam S.M., Shui G. (2020). Integration of lipidomics and metabolomics for in-depth understanding of cellular mechanism and disease progression. J. Genet. Genomics.

[52] Yu H., Villanueva N., Bittar T., Arsenault E., Labonté B., Huan T. (2020). Parallel metabolomics and lipidomics enables the comprehensive study of mouse brain regional metabolite and lipid patterns. Anal. Chim. Acta.

[53] Wang J., Zeng Y., Song J., Zhu M., Zhu G., Cai H., Chen C., Jin M., Song Y. (2023). Perturbation of arachidonic acid and glycerolipid metabolism promoted particulate matter-induced inflammatory responses in human bronchial epithelial cells. Ecotoxicol. Environ. Saf..

[54] Giovacchini G., Chang M.C., Channing M.A., Toczek M., Mason A., Bokde A.L., Connolly C., Vuong B.-K., Ma Y., Der M.G. (2002). Brain incorporation of [11C] arachidonic acid in young healthy humans measured with positron emission tomography. J. Cereb. Blood Flow. Metab..

[55] Zhuo C., Hou W., Tian H., Wang L., Li R. (2020). Lipidomics of the brain, retina, and biofluids: From the biological landscape to potential clinical application in schizophrenia. Transl. Psychiatry.

[56] Arici M.K., Tuncbag N. (2024). Unveiling hidden connections in omics data via pyPARAGON: An integrative hybrid approach for disease network construction. Brief. Bioinform..

[57] Wozniak J.M., Li W., Governa P., Chen L.Y., Jadhav A., Dongre A., Forli S., Parker C.G. (2024). Enhanced mapping of small-molecule binding sites in cells. Nat. Chem. Biol..

[58] Evangelista L., Zattoni F., Cassarino G., Artioli P., Cecchin D., Dal Moro F., Zucchetta P. (2021). PET/MRI in prostate cancer: A systematic review and meta-analysis. Eur. J. Nucl. Med. Mol. Imaging.

[59] Gaca-Tabaszewska M., Bogusiewicz J., Bojko B. (2022). Metabolomic and lipidomic profiling of gliomas—A new direction in personalized therapies. Cancers.

[60] Piazza I., Beaton N., Bruderer R., Knobloch T., Barbisan C., Chandat L., Sudau A., Siepe I., Rinner O., de Souza N. (2020). A machine learning-based chemoproteomic approach to identify drug targets and binding sites in complex proteomes. Nat. Commun..

[61] Karshikoff B. (2024). Why PNI scientists need to engage in exploratory hypothesis-generating biomarker studies. Brain Behav. Immun. Health.

[62] Young A.L., Oxtoby N.P., Ourselin S., Schott J.M., Alexander D.C. (2015). A simulation system for biomarker evolution in neurodegenerative disease. Med. Image Anal..

[63] van der Veen R., Bonapersona V., Joëls M. (2020). The relevance of a rodent cohort in the Consortium on Individual Development. Dev. Cogn. Neurosci..

[64] Fahlström A., Yu Q., Ulfhake B. (2011). Behavioral changes in aging female C57BL/6 mice. Neurobiol. Aging.

[65] Xie L., Das S.R., Li Y., Wisse L.E.M., McGrew E., Lyu X., DiCalogero M., Shah U., Ilesanmi A., Denning A.E. (2025). A multi-cohort study of longitudinal and cross-sectional Alzheimer’s disease biomarkers in cognitively unimpaired older adults. Alzheimers Dement..

[66] Xu Y., Liu S., Zhou Z., Qin H., Zhang Y., Zhang G., Ma H., Han X., Liu H., Liu Z. (2025). Integrated multi-omics analysis revealed the molecular networks and potential targets of cellular senescence in Alzheimer’s disease. Hum. Mol. Genet..

[67] Gupta H., Suk K.T., Kim D.J. (2021). Gut Microbiota at the Intersection of Alcohol, Brain, and the Liver. J. Clin. Med..

[68] Yan M., Man S., Sun B., Ma L., Guo L., Huang L., Gao W. (2023). Gut liver brain axis in diseases: The implications for therapeutic interventions. Signal Transduct. Target. Ther..

[69] He X., Hu M., Xu Y., Xia F., Tan Y., Wang Y., Xiang H., Wu H., Ji T., Xu Q. (2025). The gut-brain axis underlying hepatic encephalopathy in liver cirrhosis. Nat. Med..

[70] Bauer E.E., Shoeman A., Buhr T.J., Daniels K.M., Lyte M., Clark P.J. (2021). Voluntary binge-patterned alcohol drinking and sex-specific influences on monoamine-related neurochemical signatures in the mouse gut and brain. Alcohol. Clin. Exp. Res..

[71] Mohajeri M.H., La Fata G., Steinert R.E., Weber P. (2018). Relationship between the gut microbiome and brain function. Nutr. Rev..

[72] Tanaka M. (2025). From Serendipity to Precision: Integrating AI, Multi-Omics, and Human-Specific Models for Personalized Neuropsychiatric Care. Biomedicines.

[73] Sheikh M., Qassem M., Kyriacou P.A. (2021). Wearable, Environmental, and Smartphone-Based Passive Sensing for Mental Health Monitoring. Front. Digit. Health.

[74] Smith D.G. (2018). Digital phenotyping approaches and mobile devices enhance CNS biopharmaceutical research and development. Neuropsychopharmacology.

[75] Psaltos D., Chappie K., Karahanoglu F.I., Chasse R., Demanuele C., Kelekar A., Zhang H., Marquez V., Kangarloo T., Patel S. (2019). Multimodal Wearable Sensors to Measure Gait and Voice. Digit. Biomark..

[76] Chen S., Lach J., Lo B., Yang G.Z. (2016). Toward Pervasive Gait Analysis With Wearable Sensors: A Systematic Review. IEEE J. Biomed. Health Inf..

[77] Tanaka M. (2024). Beyond the boundaries: Transitioning from categorical to dimensional paradigms in mental health diagnostics. Adv. Clin. Exp. Med..

[78] Kotov R., Krueger R.F., Watson D. (2018). A paradigm shift in psychiatric classification: The Hierarchical Taxonomy Of Psychopathology (HiTOP). World Psychiatry.

[79] Cuthbert B.N. (2014). The RDoC framework: Facilitating transition from ICD/DSM to dimensional approaches that integrate neuroscience and psychopathology. World Psychiatry.

[80] Lilienfeld S.O., Treadway M.T. (2016). Clashing Diagnostic Approaches: DSM-ICD Versus RDoC. Annu. Rev. Clin. Psychol..

[81] McGorry P.D., Hickie I.B., Kotov R., Schmaal L., Wood S.J., Allan S.M., Altınbaş K., Boyce N., Bringmann L.F., Caspi A. (2025). New diagnosis in psychiatry: Beyond heuristics. Psychol. Med..

[82] Ghosh S., Zhao X., Alim M., Brudno M., Bhat M. (2025). Artificial intelligence applied to ‘omics data in liver disease: Towards a personalised approach for diagnosis, prognosis and treatment. Gut.

[83] Jiang L., Xu C., Bai Y., Liu A., Gong Y., Wang Y.P., Deng H.W. (2024). Autosurv: Interpretable deep learning framework for cancer survival analysis incorporating clinical and multi-omics data. NPJ Precis. Oncol..

[84] Paolini A., Baldassarre A., Bruno S.P., Felli C., Muzi C., Ahmadi Badi S., Siadat S.D., Sarshar M., Masotti A. (2022). Improving the Diagnostic Potential of Extracellular miRNAs Coupled to Multiomics Data by Exploiting the Power of Artificial Intelligence. Front. Microbiol..

[85] Danek B.P., Makarious M.B., Dadu A., Vitale D., Lee P.S., Singleton A.B., Nalls M.A., Sun J., Faghri F. (2024). Federated learning for multi-omics: A performance evaluation in Parkinson’s disease. Patterns.

[86] Westerlund A.M., Hawe J.S., Heinig M., Schunkert H. (2021). Risk Prediction of Cardiovascular Events by Exploration of Molecular Data with Explainable Artificial Intelligence. Int. J. Mol. Sci..

[87] Yan C., Yan H., Liang W., Yin M., Luo H., Luo J. (2024). DP-SSLoRA: A privacy-preserving medical classification model combining differential privacy with self-supervised low-rank adaptation. Comput. Biol. Med..

[88] Sarmadi A., Fu H., Krishnamurthy P., Garg S., Khorrami F. (2022). Privacy-Preserving Collaborative Learning Through Feature Extraction. IEEE Trans. Dependable Secur. Comput..

[89] Chen M., Guo K., Ding Y., Liu W., Yu R., Zhang L., Hu Y., Wu Y., Zhang R. (2024). Vagus nerve stimulation modulating the directed brain network of patients with drug-resistant epilepsy. Biomed. Signal Process. Control.

[90] Ryvlin P., Rheims S., Hirsch L.J., Sokolov A., Jehi L. (2021). Neuromodulation in epilepsy: State-of-the-art approved therapies. Lancet Neurol..

[91] Gouveia F.V., Warsi N.M., Suresh H., Matin R., Ibrahim G.M. (2024). Neurostimulation treatments for epilepsy: Deep brain stimulation, responsive neurostimulation and vagus nerve stimulation. Neurotherapeutics.

[92] McVey Neufeld K.A., Bienenstock J., Bharwani A., Champagne-Jorgensen K., Mao Y., West C., Liu Y., Surette M.G., Kunze W., Forsythe P. (2019). Oral selective serotonin reuptake inhibitors activate vagus nerve dependent gut-brain signalling. Sci. Rep..

[93] Hwang Y.K., Oh J.S. (2025). Interaction of the Vagus Nerve and Serotonin in the Gut-Brain Axis. Int. J. Mol. Sci..

[94] Kaizer A.M., Hobbs B.P., Koopmeiners J.S. (2018). A multi-source adaptive platform design for testing sequential combinatorial therapeutic strategies. Biometrics.

[95] Coalition A.P.T. (2019). Adaptive platform trials: Definition, design, conduct and reporting considerations. Nat. Rev. Drug Discov..

[96] Li J., Chen L., Sun H., Zhan M., Laurent R., Mignani S., Majoral J.P., Shen M., Shi X. (2023). Cationic phosphorus dendron nanomicelles deliver microRNA mimics and microRNA inhibitors for enhanced anti-inflammatory therapy of acute lung injury. Biomater. Sci..

[97] Clarke N.W., James N.D. (2023). How to Compose Platform Trials. Eur. Urol. Focus..

[98] Misra B.B., Langefeld C.D., Olivier M., Cox L.A. (2018). Integrated Omics: Tools, Advances, and Future Approaches. J. Mol. Endocrinol..

[99] Chen T., Abadi A., Cao K., Tyagi S. (2021). multiomics: A user-friendly multi-omics data harmonisation R pipeline. F1000Research.

[100] Santra S., Kukreja P., Saxena K., Gandhi S., Singh O.V. (2024). Navigating regulatory and policy challenges for AI enabled combination devices. Front. Med. Technol..

[101] Exley A.R., Rantell K., McBlane J. (2020). Clinical development of cell therapies for cancer: The regulators’ perspective. Eur. J. Cancer.

[102] Liu X., Cruz Rivera S., Moher D., Calvert M.J., Denniston A.K. (2020). Reporting guidelines for clinical trial reports for interventions involving artificial intelligence: The CONSORT-AI extension. Lancet Digit. Health.

[103] Ehidiamen A., Oladapo O. (2024). Enhancing ethical standards in clinical trials: A deep dive into regulatory compliance, informed consent, and participant rights protection frameworks. World J. Biol. Pharm. Health Sci..

